# Synthetic paclitaxel-octreotide conjugate reversing the resistance of A2780/Taxol to paclitaxel in xenografted tumor in nude mice

**DOI:** 10.18632/oncotarget.13120

**Published:** 2016-11-04

**Authors:** Xi Chen, Xiao-Yu Zhang, Yang Shen, Li-Li Fan, Mu-Lan Ren, Yong-Ping Wu

**Affiliations:** ^1^ Department of Obstetrics and Gynaecology, Zhongda Hospital, School of Medicine, Southeast University, Nanjing 210009, China; ^2^ Jiangsu Provincial Institute of Materia Medica, Nanjing 210009, China

**Keywords:** ovarian cancer, paclitaxel, somatostatin, conjugate, resistance

## Abstract

Peptide hormone-based targeted therapy to tumors has been studied extensively. Our previous study shows that somatostatin receptor expresses high level on drug-resistant human ovarian cancer. The paclitaxel-octreotide conjugate (POC) exhibits enhanced growth inhibition, as well as reduced toxicity, in paclitaxel-resistant human ovarian cancer cells. The aim of this study was to investigate the effect of targeted cytotoxicity and potential reversal mechanism of resistance in paclitaxel-resistant human ovarian cancer cells xenografted into nude mice. The SSTR2 shows higher expression levels in tumor tissue. Moreover, fluorescein-labeled POC displays favorable targeting in tumor cells. POC presents the perfect efficacy in inhibiting tumor growth and exerts lower or no toxic effects on normal tissues. Real-time PCR and Western Blotting has demonstrated that the mRNA and protein expressions of SSTR2 in POC group were significantly higher, while MDR1, α-tubulin, βIII-tubulin, VEGF and MMP-9 were significantly lower than in the other treatment groups and controls. Combined with the previous study *in vitro*, this study evaluates an effective approach on the treatment of paclitaxel-resistant ovarian cancer which expresses somatostatin receptor SSTR. Our investigation has also revealed the possible molecular mechanism of POC in treating the ovarian cancer, and therefore, provided a theoretical basis for the clinical application of this newly-invented compound.

## INTRODUCTION

Ovarian cancer is the leading cause of deaths among female gynecological malignancies. Due to the indolence at the early stage and lack of early diagnostic methods, approximately 70% of patients are diagnosed at advanced stage (stage III and IV) [1–2]. Cytoreductive surgery, followed by systematic chemotherapy of platinum and paclitaxel, is adapted as the standard treatment for advanced epithelial ovarian cancer. Even though the treatment is initially effective to 80% patients, recurrent cancer with paclitaxel resistance is inevitable in many cases, resulting in subsequent failure of chemotherapy [3–4]. Moreover, available evidence indicates that the effect of second-line chemotherapy is compromised due to heterogeneity in populations [5]. Therefore, exploring new anticancer drugs and reversing drug resistance are particularly important.

Natural somatostatin (SST), also known as somatotropin release-inhibiting factor (SRIF), is a cyclic peptide, with a wide range of cellular functions, such as inhibiting the secretion of various hormones, blocking cell proliferation and promoting apoptosis. As one of the short peptides, somatostatin is an optional agent in receptor targeting radiotherapy and chemotherapy. This is based on its ease of synthesis and optimization, which relatively small molecular weight, fast cycling clearance, and good tissue penetration ability [6–8]. The biological effects of somatostatin are mediated by targeting the five somatostatin receptors (SSTR) expressed on cell membranes [9–10]. Somatostatin receptors, which are a group of mediators for somatostatin function, are distributed extensively in neuroendocrine tumors as well as carcinomas such as breast cancer, colon cancer, non-small cell lung cancer, ovarian cancer and cervical cancer [11–12]. These observations raise the possibility of SST and its analogue as potential tumor therapeutic molecules. Compared to natural somatostatin, synthetic somatostatin analogues (SSTA) like lanreotide, octreotide and vapreotide show more advantages including longer half-life, metabolic stability, more specificity and efficiency [13–15]. Studies have shown that SSTAs not only inhibit tumor cell proliferation *in vitro*, but also suppress the growth of solid tumors *in vivo*.

One of the five somatostatin receptors, SSTR2, can be detected to be expressed in tumor, and SSTA has much higher binding affinity to SSTR2 in tumor tissues than in normal tissues [16–17]. SSTR2 can mediate the biological effects of somatostatin as the latter can block the cell proliferation, promote apoptosis and inhibit the secretion of various hormones [9–10]. Furthermore, recent studies have found that the expression of SSTR2 in ovarian cancer tissue is quite high and SSTA has the capacity to inhibit and reverse paclitaxel resistance from multiple pathways [18–24]. Some researchers conjugated SSTA (RC121, RC160) with paclitaxel into different targeted drugs and found that the conjugate increased intracellular concentration of paclitaxel, which was cytotoxic to breast cancer cells and its stem cells [25].

Octreotide (OCT) is the most widely applied representative of SSTA, and can inhibit the growth of various carcinomas including gastrointestinal tumors, breast cancer, besides neuroendocrine tumors and leukemia, through targeted binding to somatostatin receptor on tumor cells [26–28]. OCT also displays a high affinity for SSTR2, induces the activation of tyrosine phosphatase and inhibits the proliferation of SSTR2 expressing cells [29]. Our previous studies confirmed that OCT, in combination with cisplatin, can effectively inhibit the growth of SKOV3/DDP cisplatin-resistant human ovarian cancer, promote its apoptosis and reverse its resistance to cisplatin [30–31]. We have also detected that A2780/Taxol paclitaxel-resistant human ovarian cancer cell expresses SSTR2 and therefore, the synthetic POC could exert its functions in a dose and time-dependent manner via its specific binding to cell surface [32].

Some other studies indicate that chemotherapy based on SSTR-targeting has higher efficiency and lower toxicity in comparison to traditional chemotherapy. Some scholars conjugated doxorubicin or gemcitabine with SSTA and discovered that the conjugates had cytotoxic effect specifically on tumor tissues which express SSTR but lower toxicity to normal tissues in animal models. Furthermore, even in tumors with relatively low densities of SSTR expression, the conjugates are more effective and less toxic [33–35].

Based on the previous findings, this study aims to investigate the targeted effectiveness of POC and its mechanism on reversing the resistance of paclitaxel in A2780/Taxol ovarian cancer xenografted in the animal model.

## RESULTS

### Inhibition of tumor growth

**Figure 1:**
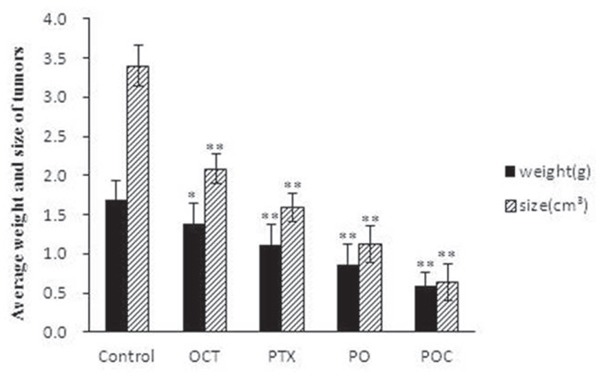
Average weight and size of xenografted tumors Mice were treated with paclitaxel (PTX), octreotide (OCT), paclitaxel combined with octreotide (PO), paclitaxel-octreotide conjugate (POC) or saline only at the indicated concentration. Compared to the other treatment, POC exhibited an enhanced inhibitory effect on tumor growth (*P*<0.05) (* *P*< 0.05 vs Control; ** *P*< 0.01 vs Control).

All the xenografted tumors grew up to about 1cm^3^ in size after 10 days following subcutaneous injection of A2780/Taxol ovarian cancer cells. At this time, no symptoms of apathy, diarrhea or poor vitality was observed in all the tested mice. After four weeks following the first administration, the average tumor weight and size in any treatment group were significantly lower than those in the control group (*P* <0.05). In comparison with the octreotide group and paclitaxel group, the average weight and size of tumor in PO group (0.86 gm, 1.12 cm^3^) were even lower and smaller (*P*<0.05). In addition, POC presented the perfect efficacy in tumor growth inhibition with a significantly decreased average weight and size of tumor (0.59 gm, 0.64 cm^3^) than any other treatments (*P*<0.05; Table 1, Figure 1).

**Table 1:** Average weight and size of tumors in different groups

Groups	weight(g)	size(cm^3^)	inhibition rate(%)
Control	1.70±0.24	3.40±0.26	—
Octreotide^a^	1.38±0.27	2.08±0.19	39%
Paclitaxel^a^^,^^b^	1.11±0.26	1.61±0.18	53%
PO^a^^,^^b^^,^^c^	0.86±0.26	1.12±0.23	67%
POC^a^^,^^b^^,^^c^^,d^	0.59±0.18	0.64±0.24	81%

a *P*<0.05 vs Control;

b *P*<0.05 vs Octreotide group;

c *P*<0.05 vs Paclitaxel group;

*P*<0.05 vs PO group.

### Targeting ability of POC *in vivo*

**Figure 2:**
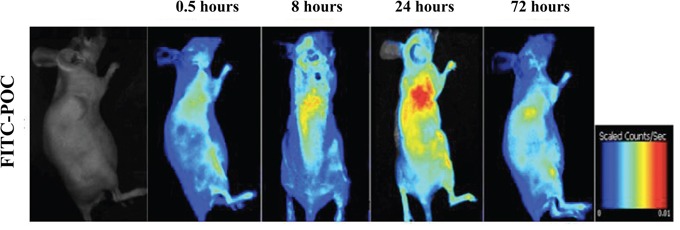
Analysis of specific binding of POC in xenografted tumor The targeting ability analysis of POC was performed by administrating of fluorescein-labeled paclitaxel-octreotide conjugate (FITC-POC). A significant aggregation of fluorescence into the xenografted tumor was observed at 8 hours with reaching the peak at 24 hours. After administration for 72 hours, fluorescence was still visible in tumor site.

When the xenografted tumor grew up to about 1cm^3^, intravenous injection of fluorescence labeled paclitaxel-octreotide conjugate (FITC-POC) was performed in selected nude mice. 30 minutes after the administration, the fluorescent signals were initially observed in tumor site, and the fluorescence intensity was gradually increased during the prolonged observation (Figure 2A). A significant aggregation of fluorescence into the xenografted tumor at 8 hours reaching the peak at 24 hours indicated a favorable targeting of POC. After administration for 72hours, part of the fluorescence was still visible within the tumor site. Besides, mild fluorescent signals could be found aggregated in liver and kidney at each time point. These findings suggested that POC presented specific targeting to paclitaxel-resistant human ovarian cancer cells in nude mice and was probably metabolized by the liver and excreted by the kidneys.

### Histology and SSTR2 expression in xenografted tumor

**Figure 3:**
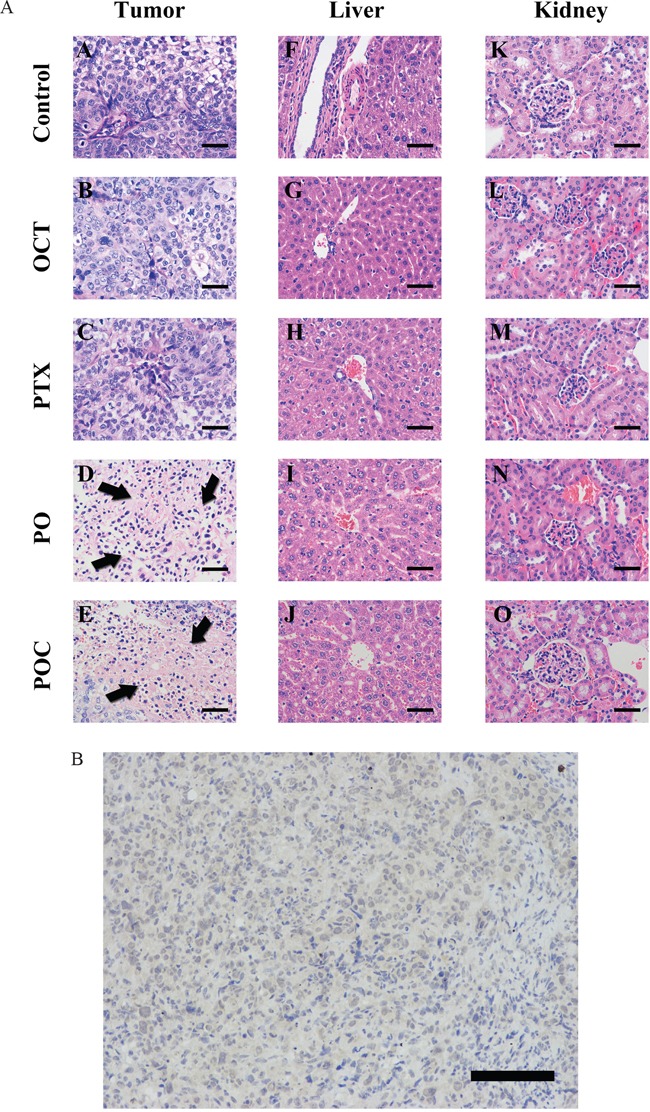
**A.** Histolog of tumor, liver, kidney by HE staining. Pathological examination was performed by HE staining. Various degrees of necrosis, inflammatory cell infiltration and fibrosis, as well as cell apoptosis, evidenced by karyorrhexis and pyknoses were observed in PO group and POC group (A, B, C, D and E; arrowheads). Tissue injury of liver (F, G, H, I and J) and kidney (K, L, M, N and O) tissues were not observed in all treated mice (magnification ×400, scale bar =200μm). **B.** Expression of SSTR2 in xenografted tumor. Immunohistochemistry with sections of xenografted tumor was detected under high power microscopic view. Brown granular particles were observed gathering on cell membrane surface and surrounding the cytoplasm, indicating SSTR2 expression of xenografted tumor was positive (magnification ×200, scale bar =400μm).

The tumors showed the same morphology as the original serous ovarian cancer by using HE stain. POC group and PO group displayed more necrosis of tumor tissues than paclitaxel group and octreotide group (Figure 3A). In addition, these morphology effects of chemotherapy were not observed in the non-targeted organs, such as liver, kidney, spleen and the tails of the treated mice (Supplemental figure 2). Therefore, it is suggested POC had lower or no toxicity to tissues outside of xenografts. SSTR2 expression in tumor tissues was detected by IHC using the monoclonal antibody against SSTR2. SSTR2 showed cell membrane expression in our study, as described in previous study (Figure 3B).

### Expression of SSTR2, MDR1, VEGF, MMP-9, Tubα1a and Tubβ3 mRNA

**Figure 4:**
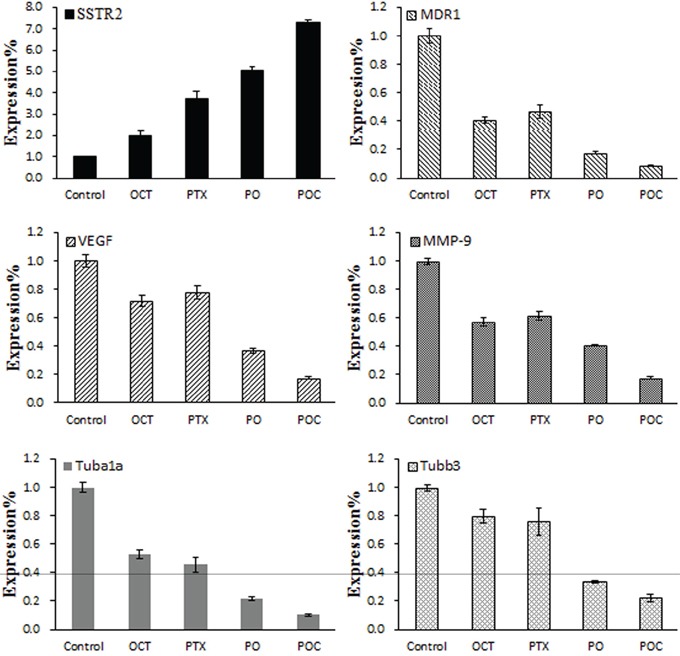
Effect of POC on the mRNA expression of SSTR2, MDR1, VEGF, MMP-9, Tubα1a and Tubβ3 in tumor tissues SSTR2, MDR1, VEGF, MMP-9, Tubα1a and Tubβ3 mRNA expressions were determined following each treatment by real-time PCR. SSTR2 mRNA expression was higher, but MDR1, VEGF, MMP-9, Tubα1a and Tubβ3 mRNA expression were lower in POC group than in the other treatment groups (*P*<0.05).

To investigate the resistance reversal mechanisms of POC, real-time PCR was performed to measure the mRNA expression levels in each treatment group. SSTR2 mRNA expression was detected in tumor tissues in all experimental groups and was higher in each treatment group than in the control group. Moreover, SSTR2 mRNA expression level in POC group was significantly higher than in the other treatment groups (*P*<0.05, Figure 4). However, the mRNA expression levels of MDR1, VEGF, MMP-9, Tubα1a and Tubβ3 in treatment groups were lower than those in the control group, and POC group has a significant lower expression than the other treatment groups (*P*<0.05). In addition, Tubα1a and Tubβ3 mRNA expression in octreotide group was higher than in paclitaxel group, but MDR1, VEGF, MMP-9 mRNA expression was lower than in paclitaxel group. The differences between each group were statistically significant (*P* <0.05).

### Expression of SSTR2, MDR1, VEGF, MMP-9, α-tubulin and βIII-tubulin protein

**Figure 5:**
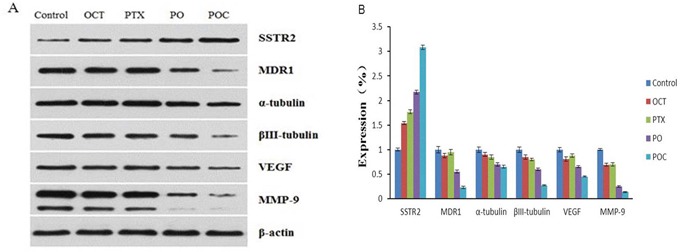
Effect of POC on the protein expression of SSTR2, MDR1, VEGF, MMP-9, α-tubulin and βIII-tubulin in tumor tissues SSTR2, MDR1, VEGF, MMP-9, α-tubulin and βIII-tubulin protein expressions by Western blot indicated that SSTR2 protein increased but MDR1, VEGF, MMP-9, α-tubulin and βIII-tubulin proteins decreased following treatment with POC (*P*<0.05) **A,B.**

Western blotting was applied to determine the protein expression levels of SSTR2, MDR1, VEGF, MMP-9, α-tubulin and βIII-tubulin after each treatment. The protein level of SSTR2 was in accordance with the mRNA expression. Namely, SSTR2 protein expressions in all treatment groups were higher than in the control group and POC group was significantly higher than the other treatment groups. Furthermore, MDR1, VEGF, MMP-9, α-tubulin and βIII-tubulin protein expressions in each treatment group were lower in comparison with those in the control group, and levels of POC group were significantly lower than those from the other treatment groups (*P* <0.05). It is worth noting that, the protein expression levels of MDR1, VEGF, MMP-9 in octreotide group were lower than those in paclitaxel group, and α-tubulin, βIII-tubulin were still higher than paclitaxel group. The differences between each group were statistically significant (*P* <0.05, Figure 5).

## DISCUSSION

Chemotherapeutic regimens based on paclitaxel and platinum combination are currently used for the treatment of patients with advanced ovarian cancer. However, due to the emergence of drug resistance, tumor relapse happens even with chemotherapy and usually leads to treatment failure. More investigations thus emphasize on the paclitaxel resistance and the development of new therapeutic agents that can overcome this resistance.

Peptide receptors are potential targets for the molecular therapies, as represented by somatostatin receptor [36]. The synthetic somatostatin analogue, octreotide, has a longer half-life than native somatostatin, and is effective in inhibition of multiple tumors such as neuroendocrine tumors, gastrointestinal cancer, breast cancer and leukemia [26–28].

Our previous study *in vitro* using the human ovarian cancer cell line found that paclitaxel-resistant cancer cells express high levels of SSTR2, and POC treatment could lead to more cell apoptosis when compared to cells treated with paclitaxel alone. Underlying cause for the difference is possibly the specific targeting of octreotide to SSTR2, which is distributed at high density on tumor cell surface [32]. Here we reported the first *in vivo* study using the nude mice. As in previous study, the high level expression of SSTR2 in xenografted paclitaxel-resistant ovarian tumor was confirmed by IHC, RT-PCR and western blot.

Previous *in vitro* research found that POC reversed chemotherapy paclitaxel-resistance through reducing the protein levels of SSTR2. However, the *in vivo* results represented in this article suggested that SSTR2 expression in POC treatment was significantly higher than in other treatment at both mRNA and protein levels. These observations may indicate that the reversal effect of POC might be associated with the increased the expression of SSTR2. The discrepancy of SSTR2 observed in Hua group between short-term and long-term treatment of Hepatocellular carcinoma using octreotide can explain the different SSTR2 expression levels in our *in vivo* and *in vitro* studies. This discrepancy could have resulted from the desensitization and internalization of SSTR2 after short-term octreotide application, and may be related to drug concentration, treatment time and microenvironment [37].

*In vivo* imaging results further showed that POC could combine with xenografted tumor specifically and POC was accumulated in tumor significantly during the prolonged observation in 24 hours. However, other organs such as the liver and the kidneys have no obvious uptake of POC. The inhomogeneous POC distribution indicated that POC can specifically target at paclitaxel-resistant human ovarian cancer cells in nude mice. In addition, the tumor weight and size of POC treatment were significantly lower than those in the other treatment group. Histologic examination also revealed that the degree of tumor necrosis and chemotherapy morphologic changes was more significant in POC group. A large lightly stained area with unclear boundary was surrounded by different amount of inflammatory cell infiltration and irregularly divided tumor cells. However, other organs showed no remarkable pathological changes. In line with our previous studies, these results further illustrated that POC could not only inhibit the proliferation of tumor cells *in vitro*, but also reverse the resistance of human ovarian cancer cell to paclitaxel and induce the better chemotherapy response of solid tumors *in vivo.* These therapeutic effects were even further optimized as reduced cytotoxicity of POC to SSTR-negative cells was observed simultaneously.

Multidrug resistance gene 1 (MDR1) is known as one of the important mechanisms causing paclitaxel resistance. Overexpression of MDR1 leads to membrane p-glycoprotein (p-gp) overexpression, which could decrease the intracellular paclitaxel concentration, and results in the reduced inhibitory effect on tumor cells by paclitaxel [31–42]. Studies have shown that, SSTA can suppress MDR1 expression and reverse drug resistance by inhibiting the PI3K/Akt signal pathway when binding to SSTR1A, SSTRR1B and SSTR2 [21]. In our study, POC treatment significantly decreased the mRNA and protein expressions of MDR1 if compared with other treatment group which was consistent with our previous study *in vitro*. Furthermore, the MDR1 expression was lower in mRNA and protein levels by octreotide treatment than paclitaxel treatment. In combination, these results suggested that the reversal mechanism of POC to paclitaxel-resistance in human ovarian cancer was possibly by reducing MDR1 expression.

Another mechanism of paclitaxel resistance may be related to abnormal expression of vascular endothelial growth factor (VEGFA, VEGF), a growth factor promoting angiogenesis and playing the critical role in tumor expansion [43]. The VEGF released by tumor tissues leads to an upregulation of MDR1 through VEGF-VEGFR signal pathway, and VEGFR antagonist can significantly inhibit the ability of drug resistance and invasion of tumors [44, 45]. Studies have demonstrated that the microvascular endothelial cells of tumor tissues sometimes express SSTR and somatostatin. These expressions could induce vasoconstriction and consequently cause circulatory disturbance to inhibit tumor growth [22–23]. This study showed that both mRNA and protein expression of VEGF after POC treatment were down-regulated, which was consistent with the result *in vitro* [32]. In addition, octreotide treatment led to a lower mRNA and protein expressions of VEGF in comparison to paclitaxel treatment. The down-regulating of VEGF expression was probably the reversal mechanisms mediated by POC.

Matrix metalloproteinases (MMPs) are demanding molecules in tumor cell invasion by degrading extracellular matrix (ECM). MMP-2 and -9 are particularly associated with the invasion of multiple gynecological maliginancies, such as cervical cancer, endometrial cancer and ovarian cancer [46]. Bergers found that MMP-9 could promote tumor angiogenesis by increasing the expression of VEGF and its receptor (VEGFR) [47]. When angiogenesis was inhibited, MMP-2 and -9 expressions are up-regulated. Inhibitors of MMPs can weaken the MMPs roles in regulation of angiogenesis by decreasing MMP-9 expression, and consequently the inhibition of tumor growth. Some other studies also revealed that up-regulation of MMP-9 expression enhanced the chemotherapeutic resistance, and SSTR2 could suppress tumor growth and metastasis by inhibition of MMP-2 and -9 expressions indirectly [24, 48]. Here we found that MMP-9 mRNA and protein expressions in tumor tissues were significantly decreased following POC treatment as compared to the other treatment and control group. Interestingly, the mRNA and protein levels of MMP-9 by octreotide treatment alone were both lower than by paclitaxel treatment alone. In summary, these findings suggested that POC reversal of the resistance of paclitaxel probably by inhibiting MMP-9 expressions in both mRNA and protein levels, and it seems that octreotide maybe more relevant to regulate the MMPs.

The anti-tumor activity of paclitaxel relies on its capability of stabilizing microtubules and the resultant interference with the G2-M transition of the cell cycle [49–50]. Thus, the altered structure of microtubules is one of the reasons for paclitaxel resistance. Fluctuation of microtubule composition expression may lead to dynamic changes in microtubule and eventually alter the tumor response to paclitaxel. The increased expression levels of α-tubulin and βIII-tubulin, mutation of βI-tubulin or down-regulation of microtubule associated proteins (MAPs) can undermine the inherent stability of microtubules and reduce the efficacy of paclitaxel [51–52]. In this study, decreased mRNA and protein expressions of α-tubulin and βIII-tubulin in tumor tissues were detected in each treatment group except the control group. Octreotide treatment showed higher α-tubulin and βIII-tubulin expression in both mRNA and protein levels than paclitaxel treatment, which was higher than octreotide combined with paclitaxel treatment. Additionally, POC treatment had the lowest expressions of α-tubulin and βIII-tubulin in all experimental groups. The alteration of tubulin expression levels may be explained by POC increasing the intracellular concentration of paclitaxel through specifically targeting octreotide on cell membrane followed by endocytosis. The endocytosis of the conjugate then concentrates the intracellular paclitaxel and promotes its inhibitory effect on α-tubulin and βIII-tubulin.

Paclitaxel-octreotide conjugate presents better effects on targeting and inhibiting paclitaxel-resistant human ovarian cancer cells in nude mice. The inhibition of POC on tumors could probably result from a series of mechanisms, including specific binding of octreotide to the membrance receptor of SSTR2, up-regulated expression of SSTR2 as well as down-regulated expressions of MDR1, α-tubulin, βIII-tubulin, VEGF and MMP-9.

## MATERIALS AND METHODS

### Peptide and chemicals

Paclitaxel (Taiji Pharmaceutical Co.Ltd., China) – octreotide (Zineng Pharmaceutical Co.Ltd., China) conjugate was synthesized in our lab, using the following steps: (1) Preparation of SDPP (N-Hydroxysuccinimido diphenyl phosphate) and paclitaxel 2' -succinate. (2) Synthesis of paclitaxel 2'-succinyl-NHS by the reaction of paclitaxel 2'-succinate with SDPP. (3) Paclitaxel - octreotide conjugate was obtained by the coupling reaction of paclitaxel 2'-succinyl-NHS with octreotide. The structure of paclitaxel - octreotide conjugate was confirmed by ESI-MS (as shown in supplemental figure 1). Fluorescein isothiocyanate (FITC) (Sigma Chemical Co.St.Louis, MO, USA) was labeled to paclitaxel - octreotide conjugate by our laboratory.

### Cell culture

Paclitaxel-resistant human ovarian cancer cell line A2780/Taxol (BogooBioTECH, Co.Ltd., China) was cultured in RPMI1640 medium (Gibco BRL Co.Ltd., USA) with 10% fetal bovine serum (FBS) (Invitrogen, USA) at 37°C in 5% CO_2_ atmosphere and 90% humidity. The log-phase cells were collected by using 0.25% trypsin (Gibco BRL Co.Ltd., USA) and then xenografted into nude mice simultaneously.

### Animals and xenografts

Female athymic nude mice (BALB/c-nu/nu) (Experimental Animal Center of Chinese Academy of Science), approximately 6-8 weeks old and weighing 18-20g were breeding in specific-pathogen-free (SPF) conditions. Xenograft models were established by subcutaneous injection of 5×10^6^ A2780/Taxol ovarian cancer cells into the right armpit of forty nude mice, and mice behavior and tumor growth were observed daily. All animal experiments were approved by Experimental Animal Ethics Committee of Southeast University.

### Growth inhibition of xenografted tumor

When the size of xenografted tumor grow up to 1cm^3^, forty healthy nude mice were chosen and averagely divided into five groups: The paclitaxel (150 nmol/kg), octreotide (150 nmol/kg), paclitaxel combined with octreotide (150 nmol/kg), paclitaxel-octreotide conjugate (150 nmol/kg) or saline only were intravenously administrated on days 1,8 and 15. Four weeks after the first administration, the mice were sacrificed to measure the size of tumors according to Steel formula tumor volume: V = ab^2^/2 (cm^3^) (a: short-track; b: long-track). The tumors were weighed in grams after stripping out the tumors, removing adjacent non-tumor soft tissue. Then the inhibitory rate of each drugs were calculated as follows: inhibitory rate = (V_control group_ −V_experimental group_)/V_control group_ × 100 %.

### Targeting ability studies

To evaluate the targeting ability of POC, we used nude mice with the tumor size larger than 1cm^3^ for the imaging experiments. The mice were intravenously injected with fluorescein-labeled paclitaxel-octreotide conjugate (FITC-POC) (150 nmol/kg). After being anesthetized with isoflurane (2-3% concentration), all mice were observed by using the automated small-animal *in vivo* imaging systems (Maestro™ Automated *In-Vivo* Imaging, USA) at multiple time points (0.5 and 8, 24 and 72hours).

### Histopathology and immunohistochemistry assay

To perform pathological examination, the tumor tissue of mice in each treated group was fixed with 4% formaldehyde solution, dehydrated, embedded in paraffin and cut into 5μm-thick serial sections. Selected sections were then stained with hematoxylin–eosin staining (HE stain). Morphological changes of tumor tissues and non-targeted organs were observed under light microscope and confocal microscope (Olympus FluoView ™ FV1000, Japan). The primary rabbit anti-mouse SSTR2 monoclonal antibody (ab134152, diluted 1:100; Abcam) was used to investigate whether SSTR2 was expressed in tumor tissues.

### Quantitative realtime-PCR and western blot analysis

The tumor tissues were cut into small pieces and homogenized into powder in Trizol reagent until the slurry was without transparent particles (100 mg: 1ml). The cell lysate was transferred into a centrifuge tube and placed at room temperature for 5min. After centrifuging at 12,000rpm, at 4°C for 10min, supernatant was carefully transferred into a new centrifuge tube. The mRNA and protein expressions of MDR1/p-gp, α-tubulin, βIII-tubulin, VEGF and MMP-9 were detected as described in manual from RT-PCR kit (Takara Biotechnology Co.Ltd., Dalian, China) and BeyoECL star (Beyotime Biotechnology, Co.Ltd., China), respectively. Specific primary antibodies (SSTR2(1:2000), MDR1(1:1000), VEGF(1:1000), MMP-9(1:1000), α-tubulin(1:1000), βIII-tubulin(1:1500), β – actin(1:1000)) were applied.

### Statistical analysis

Statistical analysis was performed using SPSS 19.0 statistical software. The significance between groups were analysed by variance (ANOVA) and q test (Student-Newman-Keuls). Data were expressed as mean ± standard deviation (SD). A *P* value of <0.05 was considered statistically significant.

## SUPPLEMENTARY FIGURES


