# Supplementary material for: Synthetic paclitaxel-octreotide conjugate reversing the resistance of A2780/Taxol to paclitaxel in xenografted tumor in nude mice
Source: Oncotarget. 2016 Nov 4;7(50):83451–61. doi: 10.18632/oncotarget.13120 (PMC5347781; doi:10.18632/oncotarget.13120)
Supplement: Supplementary file 1 [file oncotarget-07-83451-s001.pdf]

Synthetic paclitaxel-octreotide conjugate reversing the resistance of A2780/Taxol to paclitaxel in xenografted tumor in nude mice

SUPPLEMENTARY FIGURES

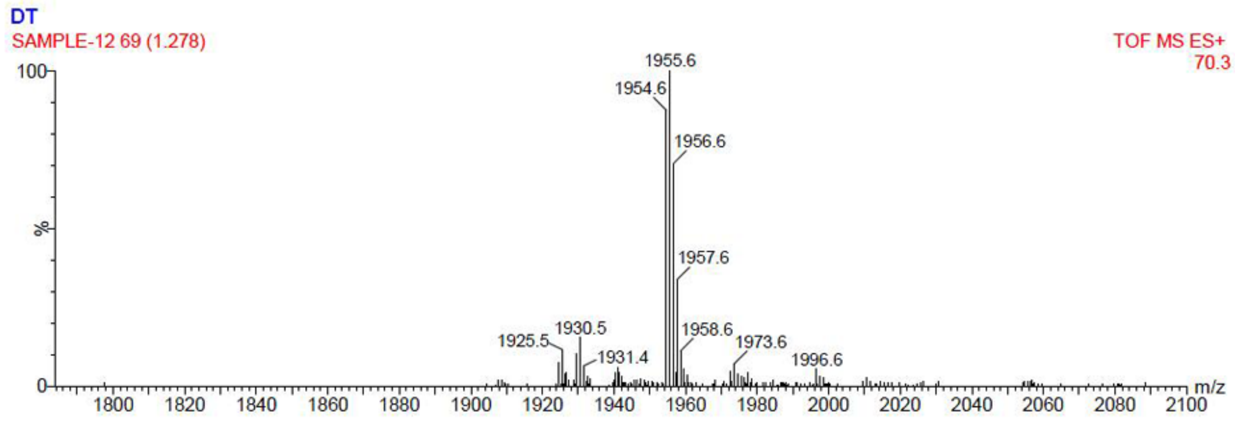

Supplementary Figure S1: Analysis of POC by ESI-MS. High Performance Liquid Chromatography (HPLC) was used to prepare a small amount of conjugate sample, which then examed by electrospray ionization mass spectrometry (ESI-MS). The conjugate was consistent with the target POC, ESI-MS (m/z):1954$[M+H]^+$.

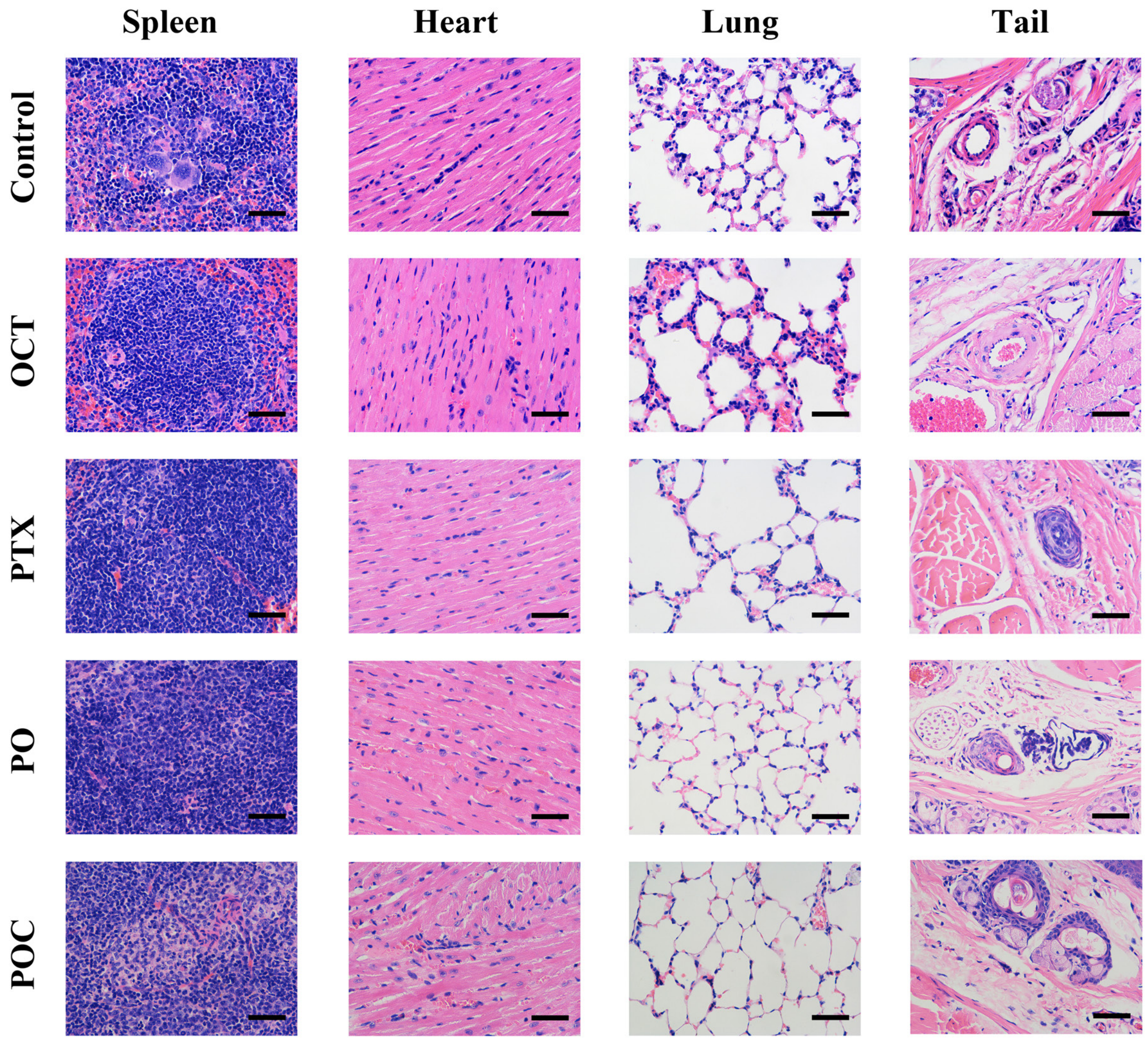

Supplementary Figure S2: Histolog of spleen, heart, lung, tail by HE staining. Pathological examination was performed by HE staining. Morphology effects like necrosis and cell apoptosis of chemotherapy were not observed in the spleen, heart, lung and tail of the treated mice in all treatment groups (magnification ×400, scale bar =200μm).
